# Method to evaluate the noise of 3D intra-oral scanner

**DOI:** 10.1371/journal.pone.0182206

**Published:** 2017-08-09

**Authors:** Alban Desoutter, Osama Yusuf Solieman, Gérard Subsol, Hervé Tassery, Frédéric Cuisinier, Michel Fages

**Affiliations:** 1 Laboratoire Bioingénierie et Nanosciences, Montpellier University, Montpellier, France; 2 Project-Team ICAR, Laboratoire d'Informatique, de Robotique et de Microélectronique de Montpellier, Centre National de la Recherche Scientifique, Montpellier University, Montpellier, France; Eberhard-Karls-Universitat Tubingen Medizinische Fakultat, GERMANY

## Abstract

In dentistry, 3D intra-oral scanners are gaining increasing popularity essentially for the production of dental prostheses.

However, there is no normalized procedure to evaluate their basic performance and enable comparisons among intra-oral scanners. The noise value highlights the trueness of a 3D intra-oral scanner and its capacity to plan prosthesis with efficient clinical precision. The aim of the present study is to develop a reproducible methodology for determining the noise of an intra-oral scanner. To this aim, and as a reference, an ultra-flat and ultra-smooth alumina wafer is used as a blank test. The roughness is calculated using an AFM (atomic force microscope) and interferometric microscope measurements to validate this ultra-flat characteristic. Then, two intra-oral scanners (Carestream CS3500 and Trios 3Shape) are used. The wafer is imaged by the two intra-oral scanners with three different angles and two different directions, 10 times for each parameter, given a total of 50 3D-meshes per intra-oral scanner. RMS (root mean square), representing the noise, is evaluated and compared for each angle/direction and each intra-oral scanner, for the whole mesh, and then in a central ROI (region of interest). In this study, we obtained RMS values ranging between 5.29 and 12.58 micrometers. No statistically significant differences were found between the mean RMS of the two intra-oral scanners, but significant differences in angulation and orientations were found between different 3D intra-oral scanners. This study shows that the evaluation of RMS can be an indicator of the value of the noise, which can be easily assessed by applying the present methodology.

## Introduction

The first design CAD-CAM (computer-aided design and computer-aided manufacturing) concept in dentistry appeared in the 1970s, with digital image acquisition in three dimensions (3D) [1]. CAD-CAM intra-oral scanners [2] are actually the competing conventional polymer material techniques in dental offices [3]. They provide a three-dimensional mesh calculated by a software and are used by the practitioner to design a virtual prosthesis to be fabricated by machining or rapid prototyping [4]. The powder system with TiO_2_ nanoparticles can also be used to allow optimum acquisition by eliminating light reflection and transparency. However, the powder could aggregate and create blocks that are a size larger than intra-oral scanner resolution [5]. New intra-oral scanners are actually powder free [6].

For intra-oral scanners, the most difficult challenge is to reproduce reality with high fidelity. In the above-cited papers, the noise is not precisely evaluated. Error of measurement and the impact of deviation from ideal angulation of intra-oral scanners were studied, but with the whole CAD-CAM system including the production unit, which was also investigated [7] [8]. Fifteen hundred reconstructions over a period of 5 years produced with Cerec 3D CAD-CAM have revealed several ambiguities of the intra-oral scanner, due to instability of the scanner, angulation problems, and the presence of saliva or artefacts [9]. Another study of the trueness of the intra-oral scanner [10] was performed using an optical 3D scanner as a reference. The intra-oral scanner resolution and error of the system were not studied. In 2012, a study was conducted on the accuracy of three intra-oral scanners (Cerec, iTero and Lava COS) [11]. Distances obtained with intra-oral scanners were compared with an optical 3D measuring machine. The distances between this reference and the intra-oral scanners were 14.6 microns for the Lava COS and up to 81.6 μm for CEREC with large standard deviations.

A recent study [12] used an epoxy model, and the reference was provided by micro computed tomography (CT). Because the microCT pixel size was 9.21 microns, close to intra-oral scanner resolution, it could be used as a reference. Measurement repeatability was also carried out by comparing each of the scans of the same object, imaged several times.

Evaluation of intra-oral scanners’ trueness is equivalent to measuring the additional noise to the signal. The ISO 5725 norm defines trueness as “the closeness of agreement between the arithmetic mean of a large number of test results and the true or accepted reference value” [13]. The difference between the true value and the value recorded by a system is the noise, a small deviation of the signal. In imaging, noise could be considered artefact information, additional to the signal [14]. The noise could result in discrete quantification of the signal [15] or variation due to the sensor or scene variation [16]. However, there is not a normalized metrological system for determining the noise of intra-oral scanners. Indisputably, noise is a relevant parameter for properly assessing the performance of intra-oral scanners, which are medical devices subject to strict regulations. To validate this methodology, the performances of the two intra-oral scanners are evaluated by determining their noise. The CS 3500 (Carestream, Rochester, USA) and the Trios (3shape, Copenhagen, Denmark) intra-oral scanners use confocal imaging capturing methods without the need of powder [17].

## Material and method

### Material

#### Alumina wafer

Blank tests are realized using an ultra-flat alumina wafer. The wafer used in the present study is a white square 99.6% alumina wafer made by the Optics Concept Company (Paris, France). The dimensions are 75 millimetres per side and 400 micrometres in height. The flatness indicated by the company is lambda/2, corresponding to a maximum wafer peak to valley length. Lambda represents the wavelength of used light (550 nm). A Fizeau interferometer was used to control the flatness.

#### AFM

To verify the flatness of this pattern, an initial test was performed with an atomic force microscope (AFM) Nanoscope 3A Quadrex (Bruker instruments, Billerica, USA). A 30*30 μm image was recorded in tapping mode. The tip was a Nanosensors NCH (Nanosensors, Neuchatel, Switzerland). Visualizations on the surface and RMS calculation were realized with Gwyddion v.2.43 software (Czech Metrology Institute, Prague, Czech Republic).

#### Light interferometric microscope

The second measurement of wafer flatness was performed with a full-field white light interferometric microscope Photomap 3D (Fogale company, Nimes, France). The measurement was realized in topography mode with maximum intensity of fringe envelope. Data visualization and RMS calculation were realized with FOGALE 3D Viewer 2006–06 software (Fogale, Nimes, France).

#### Intra-oral intra-scanners

Two intra-oral scanners were evaluated and compared:

CS3500 intra-oral scanner

CS3500 intra-oral scanner from Carestream (Rochester, New York State, USA) is a powder-free system.

It is a confocal parallel system with a green laser light and a pinhole to select a focal plan. The light projected on the tooth is collected on a CMOS (complementary metal oxide semi-conductor) 1.3 cm sensor, transforming light into an electric signal. A motor quickly moves the focal plan and a high intensity signal is collected to reconstruct the 3D mesh. During 3D recording the intra-oral scanner can capture HD photo with 4 LED (blue, green, red, UV) for realistic tooth reconstruction.

The resolution (i.e., the size of the smallest details the camera can image) reported by the company is 35 micrometres.

The software for the CS3500 scanner is CS Restore (Carestream, Rochester, New York, USA). Version number of het scanner operating software is 6.14.7.3.

Trios 2 Pod intra-oral scanner

The Trios 2 Pod intra-oral scanner (3Shape, Copenhagen, Denmark) is a powder-free intra-oral scanner based on permanent confocal imaging. Structured light is projected on the tooth through interference fringes. The mechanical oscillation of the light is coupled with variation of the confocal plan. The signal is recorded with a charge-coupled device (CCD) sensor. The difference between the intensity of the signal in each plan determines the point position.

The resolution of the Trios scanner is not provided by the company. Trueness (i.e., closeness of the meaning of measures to the real value; 6.9 ± 0.9 μm) and precision/consistency (i.e., closeness of different measures to the meaning; 4.5 ± 0.9 μm) were evaluated in previous literature [18].

The software for the Trios scanner is 3Shape Dental designer (3Shape, Copenhagen, Denmark). Version number of het scanner operating software is 1.3.4.7.

### Method

#### Acquisition

**Fig 1:**
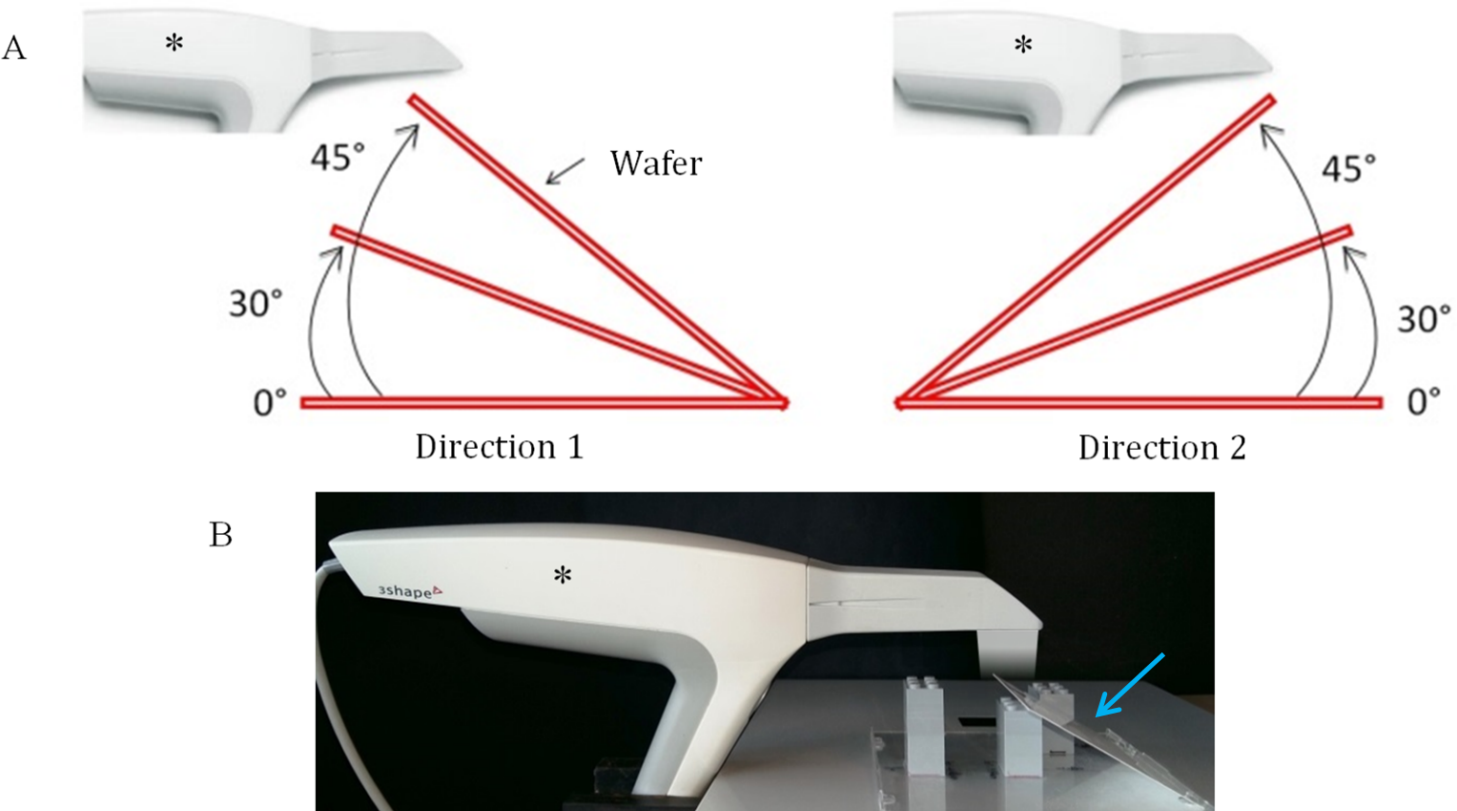
(A) blueprint of the acquisition parameters for wafer intra-oral scanner imaging. Three angulations: 0°, 30° and 45°. Two directions: “direction 1” on the left; “direction 2” on the right. (B) photo of the setup with the Trios camera (3shape, Copenhagen, Denmark); blue arrow indicates the wafer; asterisk indicates the intra-oral camera.

For each intra-oral scanner, the wafer was recorded with three angulations: 0° (wafer perpendicular to light source), 30° and 45° angles. For angles of 30° and 45°, the wafer was recorded in two directions: 1. slope in the extension of intra-oral scanner; 2. slope in the other direction, to the handle of the intra-oral scanner (Fig 1A).

Each position was recorded ten times. For each intra-oral scanner, 50 raw meshes have been created.

The intra-oral scanner was fixed with a vice, with the captor parallel to the plan where the wafer was imaged. The wafer was placed parallel to the captor for 0-degree angulation and inclined with Lego blocks for 30 and 45 degrees angulations, with a captor to wafer distance around 3 ± 0.5 cm (Fig 1B).

#### STL files extraction

STL files were imported directly from the CS3500 intra-oral scanner. The DCM files from the Trios were converted into STL files with CrossManager 2014 software v2014.4.0 (Datakit, Lyon, France).

#### Data analysis

Mesh visualization

Mesh visualization was performed using Meshlab Software (ISTI, Italian National Research Council, Italia) [19]. The meshes were systematically cropped to eliminate wafer edges.

RMS calculation

RMS, representing the noise of the intra-oral scanner, is calculated using mesh. Calculations of meshes were realized with CloudCompare v2.6.0 software (Research and Development Institute of Electricité De France EDF, Paris, France).

To visualize the noise recorded by the intra-oral scanner, the roughness function was used. A kernel size is selected, corresponding to the ball included near vertices neighbours. From these vertices, a mean plane is fitted to all the vertices using a least-square minimization, and the distances vertex-plan are calculated. With an efficient size of the kernel (20 mm ± 2 mm for the system studied), all points could be included to calculate a mean plan, corresponding to a virtual flat surface.

The mesh roughness could be considered the RMS value, which represents the mean distance of vertices to this plan.

With the null hypothesis “no noise exists”, every point of the surface should be in the plan (roughness = 0). If not, a positive value is recorded, and the calculation is realized for all vertices of the mesh.

The software creates a colour map, and each vertex is represented in a false colour, corresponding to a distance.

Every mesh was resampled a second time to select a region of interest (ROI) in the centre of the mesh to approximate the edge effect (higher roughness in the centre than on the edge of mesh). In the data treatment, distinction was realized between mesh and central ROI.

Coordinates of vertices and distances from vertices to the virtual plan were exported into Excel files. RMS is calculated by the equation:

$$R_{q}=\sqrt{\frac{1}{n}(x_{1}^{2}+x_{2}^{2}+x_{3}^{2}+\cdots +x_{n}^{2})}$$

Using Excel (Microsoft, Redmond, USA), we easily determined the RMS for each mesh.

Classification of vertices To compare the recorded meshes, each vertex was classified as a function of the distance from a virtual flat plan. For each mesh, the number of vertices localized in a determined vertex to plan distance was calculated: 0–5 μm, 5–10 μm, 10–20 μm, more than 20 μm. Different proportions of repartition of vertices and function of this distance enable us to visualize the capacity of the intra-oral scanner to record a noiseless image.

Statistical analysis

To compare different RMS results from the two intra-oral scanners, Kruskal-Wallis non-parametric tests were used.

To compare RMS values from the whole mesh and the central ROI, a Mann-Whitney rank sum test was used. For mean RMS of the two intra-oral scanners, each angle and direction was compared in pairs with a Mann-Whitney rank sum test.

## Results

### Reference RMS

**Fig 2:**
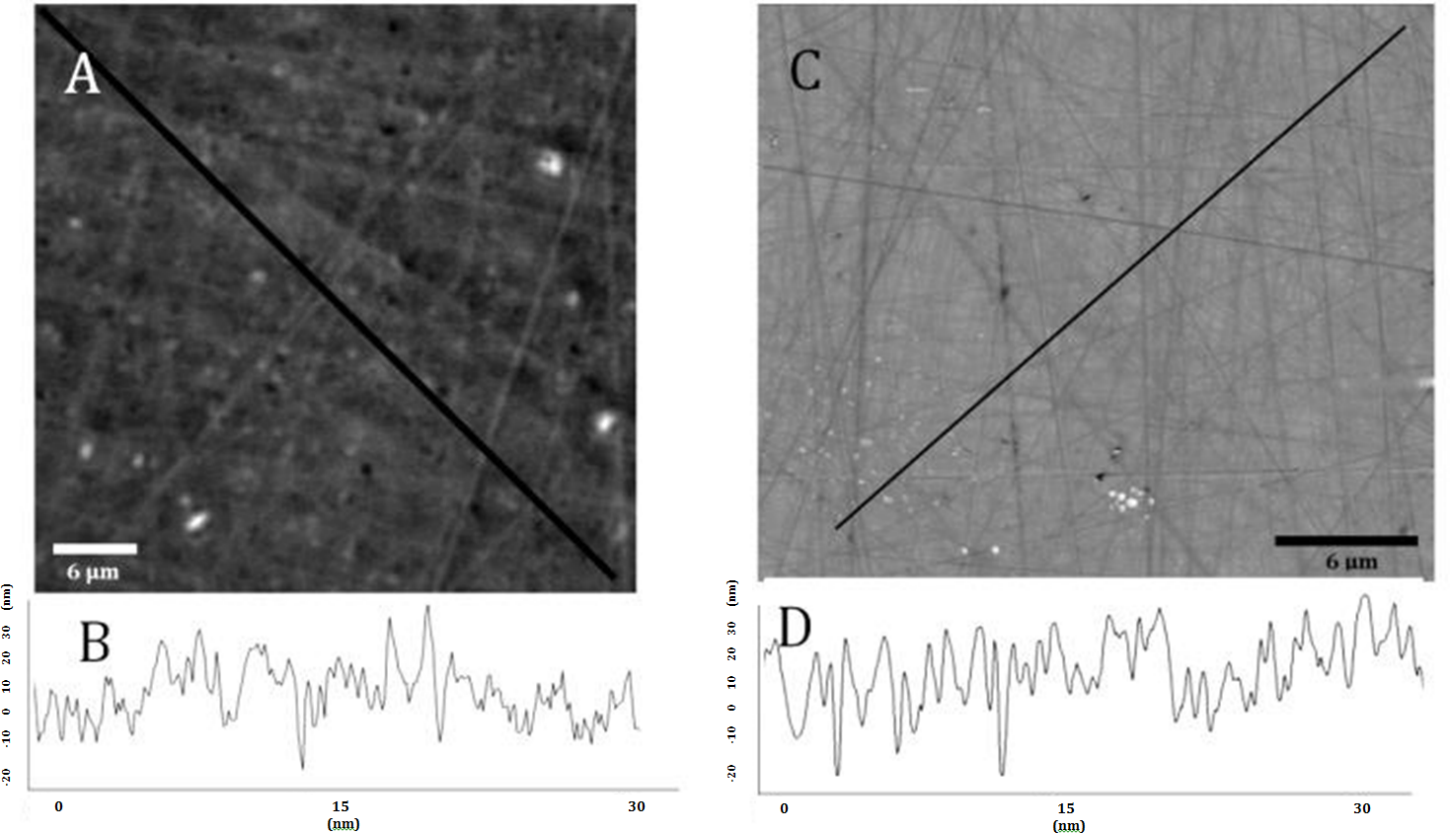
Alumina wafer. (A) interferometric microscope wafer image. (B) surface profile along the black line in A. (C) atomic force microscope wafer image. (D) surface profile along the black line in A.

A wafer was studied with an interferometric microscope (Fig 2A) on a rectangle 30 μm × 24 μm. The RMS calculated on the whole image was 9.65 nanometres.

The RMS calculated on the 900 μm^2^ surface is 17.52 nm with AFM (Fig 2C).

### Intra-oral scanners RMS

**Fig 3:**
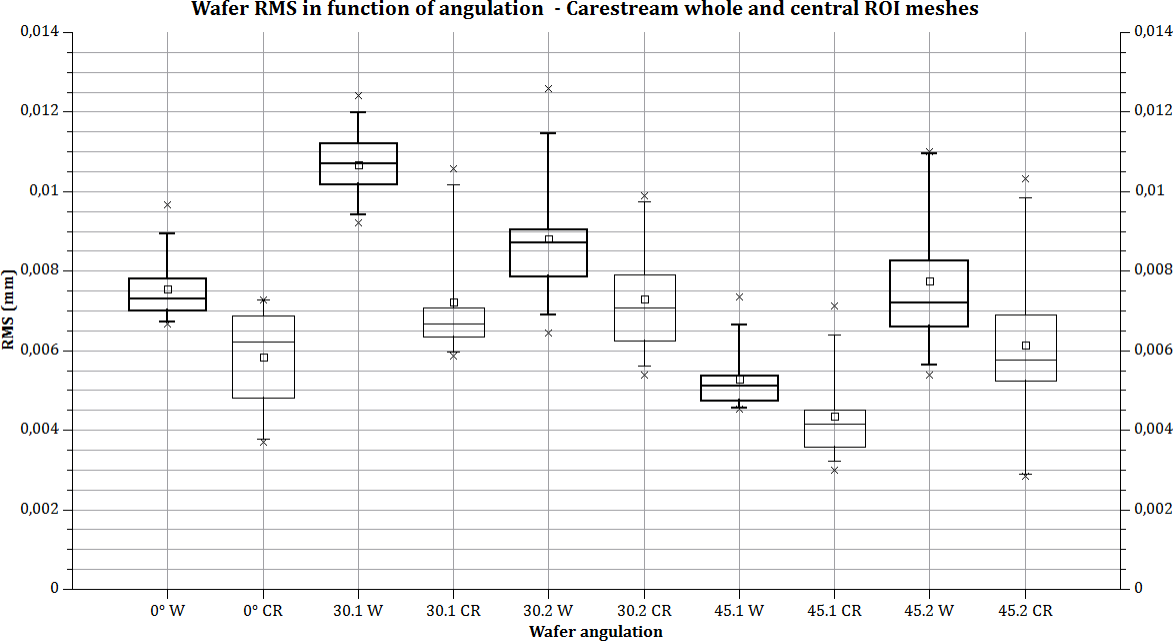
Wafer RMS calculated with CS3500. Bold lines: the whole mesh is used for RMS calculation; thin line, RMS calculated in the central part of the mesh. Box plot: rectangle: 25% to 75%. Whiskers: 5–95 range. Square: mean value. Cross: min and max value. X axis: parameter acquisition (angulation and direction 1 or 2) and calculated area (W = whole mesh; CR = central region of mesh).

**Fig 4:**
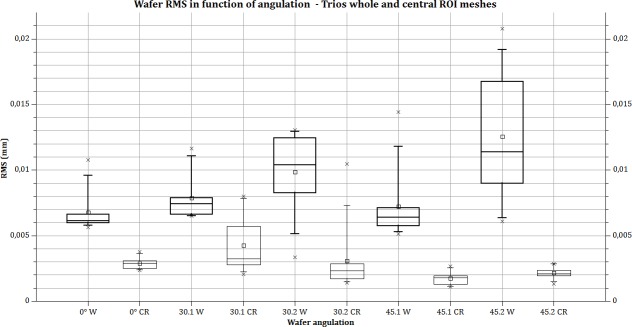
RMS calculated on wafer with CS3500. Bold lines: the whole mesh is used for RMS calculation; thin line, RMS calculated in the central part of the mesh. Box plot: rectangle: 25% to 75%. Whiskers: 5–95 range. Square: mean value. Cross: min and max value. X axis: parameter acquisition (angulation and direction 1 or 2) and calculated area (W = whole mesh; CR = central region of mesh).

**Fig 5:**
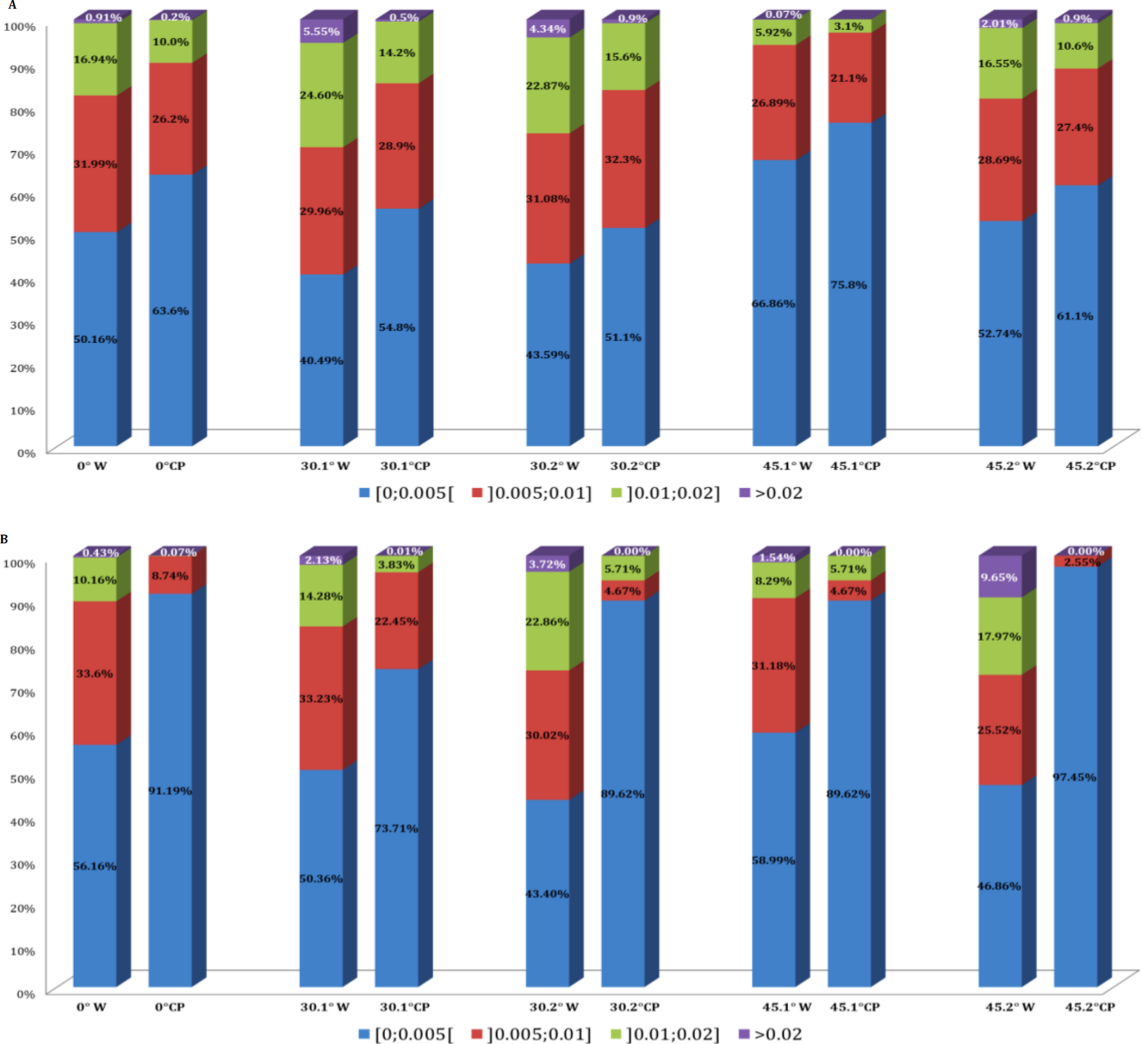
Vertices repartition of mesh stacked histogram. (A) Carestream 3500 vertices repartition. (B) Trios vertices repartition. First column: results for the whole (W) mesh. Second column: results for the central part (CP) of the mesh. Acquisition parameters are inscribed on the Y axis.

**Fig 6:**
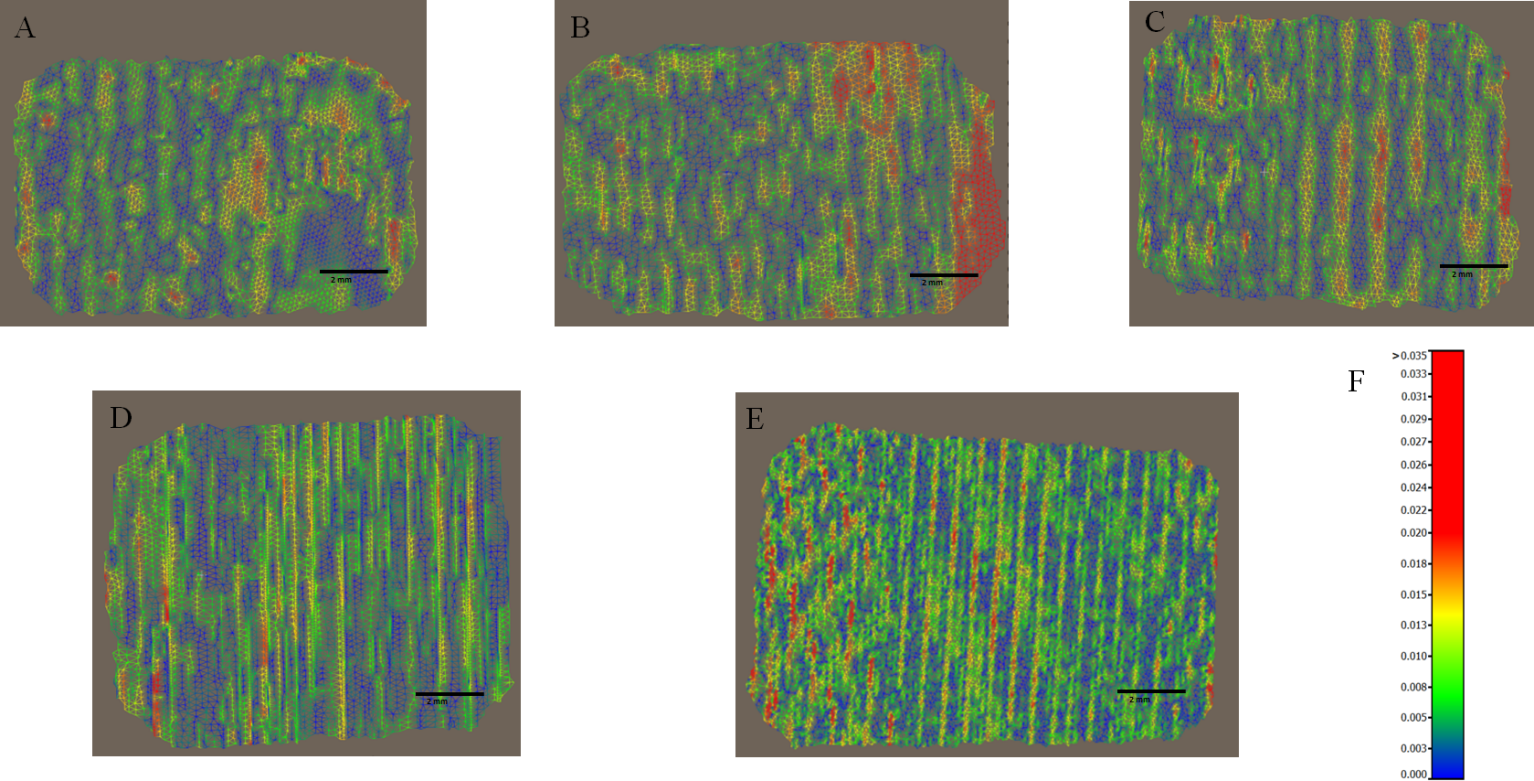
Examples of colour-mapped whole meshes imaged with a Carestream CS3500 intra-oral scanner. Distances between the mesh and the average plane. The look-up table (LUT) shows the different colours as a function of distance to the vertices. Examples of coloured map meshes of the wafer recorded with an intra-oral scanner. (A) angulation 0°. (B) angulation 30° direction 1. (C) angulation 30° direction 2. (D) angulation 45° direction 1. (E) angulation 45° direction 2. (F) look-up table corresponding to distance/colour hue.

**Fig 7:**
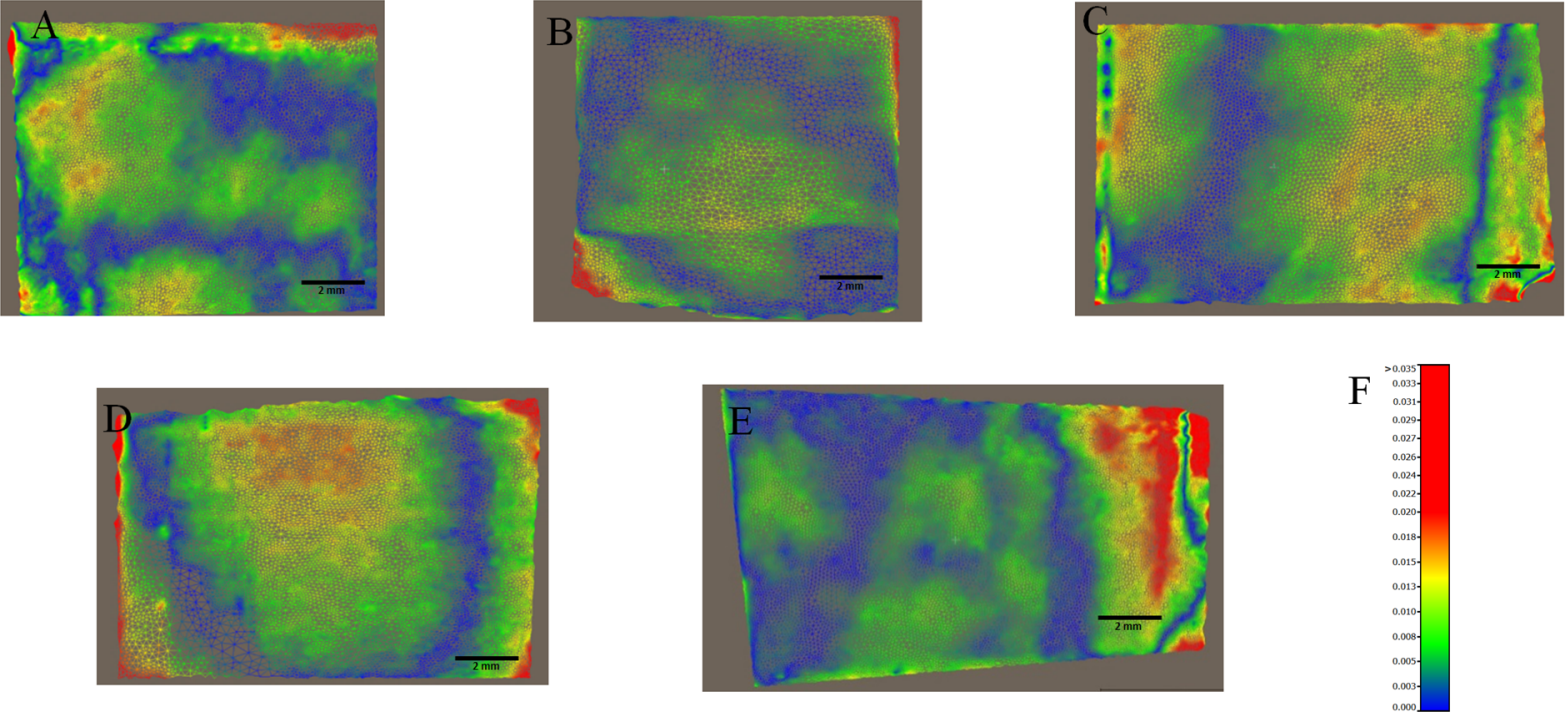
Examples of colour-mapped whole meshes imaged with the Trios 3 Shape intra-oral scanner. Distances between the mesh and the average plane. Look-up table (LUT) shows the different colours as a function of distance to the vertices. Examples of coloured map meshes of the wafer recorded with intra-oral scanners. (A) angulation 0°. (B) angulation 30° direction 1. (C) angulation 30° direction 2. (D) angulation 45° direction 1. (E) angulation 45° direction 2. (F) Look-up table corresponding to distance/colour hue.

The RMS values are calculated from meshes from intra-oral scanners. RMS values for the whole meshes varied between 5.29 (SD ±0.82) and 10.68 (SD ± 0.92) micrometres for CS3500 and between 6.79 (SD ±1.56) and 12.58 (SD ±4.95) micrometres for Trios. The different RMS results for meshes imaged by the intra-oral scanners are shown in Table 1. Figs 3 and 4 show RMS values, respectively, for CS3500 and Trios intra-oral scanners with boxplots. Fig 5 shows vertices repartition of mesh stacked histogram. Figs 6 and 7 show examples of 3D meshes coloured with a look-up table corresponding to the vertices-to-virtual plan distance.

**Table 1:** RMS results and 25–75 percentile difference for CS3500 and Trios intra-oral scanners.

	0 (a)		30.1 (b)		30.2 (c)		45.1 (d)		45.2 (e)		Total	
	Whole	Central part	Whole	Central part	Whole	Central part	Whole	Central part	Whole	Central part	Whole	Central part
**Carestream**												
**RMS (μm)**	**7.54**^**b**^^**,**^ ^**c**^	5.85	**10.68**^**a**^^**,**^ ^**d**^	7.24	**8.80**^**d**^	7.29	**5.29**^**a**^^**,**^ ^**b**^^**,**^ ^**c**^	4.36	**7.74**^**b**^	6.16	**8.01**	6.18
interquartile range	0.79	2.04	1.00	0.69	1.14	1.65	0.62	0.91	1.64	1.64	2.19	1.93
Coefficient of variation (%)	11.60	23.23	8.64	21.82	18.84	20.57	15.55	27.21	25.62	40.47	27.37	31.28
Confidence interval	0.63	0.63	0.66	0.66	1.19	1.19	0.59	0.59	1.42	1.42	1.57	1.57
**Trios 3shape**												
**RMS (μm)**	**6.79**^**c**^^**,**^ ^**e**^	2.91	**7.89**^**c**^	4.26	**9.87**^**a**^^**,**^ ^**b**^	3.08	**7.22**^**e**^	1.77	**12.58**^**a**^^**,**^ ^**d**^	2.15	**8.86**	2.83
interquartile range	0.58	0.55	1.19	2.89	4.16	1.12	1.35	0.62	7.71	0.38	3.63	1.75
Coefficient of variation (%)	22.98	16.17	22.09	51.59	31.15	86.66	37.68	29.34	39.33	21.24	41.01	61.92
Confidence interval	1.12	0.34	1.25	1.57	2.20	1.91	1.95	0.37	3.54	0.33	2.60	1.26
**p-value**	0.026	0.001	0.006	0.017	0.241*	0.003	0.007	0.001	0.025	0.001	0.841*	0.001

a, b, c, d and e superscripts indicate whether there was a significant difference. The confidence interval was calculated with an α = 0.05.

### Statistical analysis

Table 1 indicates statistical information about results on the different measures. In RMS line, up the mean value is noted the letters corresponding to significant difference for each group of measures. For example, in the first column, for Carestream CS3500 camera, (0 degree) “b, c” means significant difference between 0 degree and 30 degrees, orientation 1 and 2. Four groups of measures are significatively different for Carestream, and 5 for Trios one. Comparison between each intra-oral camera for a specific angulation or angulation/orientation is shown in the last line: P-value indicate only one non-significant difference in a group of measure (30 degree orientation 2).

## Discussion

Within the present methodology, a blank model was used to determine intra-oral scanner signal noise. The semiconductor wafer is a very flat component, as confirmed by AFM and interferometric microscopy, and can be considered an almost totally flat surface regarding intra-oral scanner resolution. A significant difference in the RMS roughness is observed between the interferometric microscope and the AFM. This result could be explained by the difference of the technics [20], but it is insignificant to scanner resolution announced by company (around 30 micrometres).

Any error of the meshes may be attributed to the convolution of optical, electronic noise and software approximation. One limitation of this study is that it was not possible to determine the origin of the noise.

To evaluate the present method, comparable to a point black assay, a zero error experiment defined as datum measurement error where the specified measured quantity value is zero was realized. The RMS found for the zero experiment is approximately 5 to 10 μm for CS3500 and 6 to 12.5 μm for Trios, regarding the intra-oral scanner light-to-wafer angulation.

For example, the RMS for the whole mesh indicates a noise on the reconstructed surface between -5 and +5 μm for the CS3500: each point of the mesh has a theoretical error of ± 5 μm in the perpendicular direction of the sample surface. These results must be compared to the necessary clinical precision. For a tooth crown, this error represents a non-significant volume.

One problem that could arise involves the brutal direction changing, for instance, on the edge of the prepared tooth.

However, in clinical practice, the variation in accuracy may have an impact on two levels. For a prepared tooth (to receive a crown, for example), the precision of the prepared surface on the cervical limit is very important and must provide the best fit between the crown and the prosthesis [21]. With the CAD-CAM process, based on optical impressions, the marginal fit obtained seems to be consistently below 100 μm [22]. In a study published in 2015, Shaefer et al. concluded that the investigated digital impression procedures demonstrated significant fit discrepancies. However, all fabricated restorations showed acceptable marginal and internal gap sizes, when considering the clinically relevant thresholds reported in the literature [23]. However, the discrepancies can be imputed not only to the accuracy of the optical impression but also to the rest of the CAD-CAM procedure [24].

If the optical print concerns an entire tooth, precision is also important to first ensure correct occlusion with the opposing teeth. To verify these contacts, most practitioners use ink paper (Shimstock) 40 microns thick and in some cases 20 microns for very fine work [25]. An accurate optical print promotes the management of these contacts. The accuracy of occlusal relationships in CAD-CAM remained an important topic for years [26]. Furthermore, this accuracy is very important for the fabrication of stereolithographic models obtained from optical impressions [27].

The first observation is about the lower repeatability of the test. Each of the tests has been conducted by the same laboratory, with the same operator using the same system, for each intra-oral scanner. The limits of repeatability, defined by the 5725 ISO norm [28] as “The value less than or equal to which the absolute difference between two test results obtained under repeatability conditions may be expected to be with a probability of 95%”, are very high for each test. The noise, represented by RMS value, is highly variable, and also non-predictable.

The influence of the operator would be highlighted by comparing results obtained with human operators scanning a model with an intra-oral scanner and models scanned with a laboratory scanner. A study published in 2014 compared the marginal fit of crowns fabricated with digital and conventional methods and concluded that the fully digital fabrication method provided a better margin fit than the conventional method, with values of 48 ±25 μm [29]. These values (48 ±25 μm) seem better than those commonly obtained with the intra-oral scanner (80 μm +/-). However, Guth JF et al, who evaluated the accuracy of different intra-oral scanners and compared them to the process of indirect digitalization, concluded that there are even differences between the tested systems, and the direct digitalization was not superior to indirect digitalization for all tested systems [30].

The noise repartition is heterogeneous. The central part of the image is always closer to the virtual plane. The more distant points from the virtual plane (>20 μm) are more likely to exist on the edge. For example, vertices between 0 and 5 micrometres from the flat plan represent 50.16% of the mesh with the CS3500, with an angulation of 0°; but for the same angulation, those vertices represent 63.6% of the central part of the mesh. This fact is observed in any angulation and direction but is higher and more notable with the Trios intra-oral scanner: vertices localized under 5 μm of the flat plan represent between 40 and 66.8%, whereas those for the central part represent between 73 and 99%. Those results demonstrate a clear edge effect: the point recorded near the limit line of the square image is far from the sample reconstruct. However, there is no measure for the moment to estimate the impact of such a problem on a tooth. We do not know at this point if the edge artefact exists for a single crown or full arch.

The statistical result demonstrates that the difference between the whole mesh and central part could be considered statistically significant. Thus, we have a clear “edge effect” with the two intra-oral scanners. This was never reported previously. The relationship between the calculated noise and intra-oral scanner angulation is not linear and is different for both intra-oral scanners. For example, for the CS3500, noise increases 41.6% from 0° to 30° (direction 1 or 2), and then decreases 50.4% from 30° to 45° (for direction 1). The calculated noise with an angulation of 45° is equivalent to 0° or less: 7.54 μm for 0°, 5.29 μm with 45° in direction 1 and 7.74 μm with 45° in direction 2.

However, with the Trios intra-oral scanner, the result is different: 6.79 μm for 0°, 7.22 μm for 45° direction 1 with a standard deviation of 2.72 μm and 12.58 μm for direction 2 with a standard deviation of 4.95 μm. A previous study from 2015 gives an RMS on a single crown approximately of 65 μm for Trios intra-oral scanner [12]. The difference from our result can be explained by the use of micro CT as a reference and of a non-flat model.

A more specific investigation must be conducted to determine if this noise signal is due to intra-oral scanner chromatic aberration, i.e., temporal coherence, or a software effect.

The limitation of our study is that we determined intra-oral scanner noise without determining the resolution. Trueness and precision also need to be studied to fully characterize the intra-oral scanner.

Moreover, other methods must be introduced in the CAD-CAM intra-oral scanner system to evaluate performance. Resolution of the intra-oral scanner, in particular, would calculate the smaller detail imaged by the system. Many domains of dental care can be improved with a well-known precision intra-oral scanner: roughness of the tooth, pre-carious diagnosis, implantology, etc.

If the wafer model is used as a method for determining the noise, an investigation must be conducted to create a reference sample for accuracy and measurement uncertainty. To confirm such a model, a round-robin test would be necessary, with sufficient specimens and laboratories to conduct a significant statistical test.

## Conclusion

The present results validate our model to determine the intra-oral camera noise and underscore the importance of establishing blank test. In that way the ultra-flat wafer is a good opto readable pattern for Trios and Carestream systems. Detected noise is significant near image border, but smaller in the center. If the wafer could be recorded for all intra-oral system, our method could become the gold standard for noise determination.
